# Comparative Evaluation of the Penetration Depth into Dentinal Tubules of Three Endodontic Irrigants

**DOI:** 10.3390/ma14195853

**Published:** 2021-10-06

**Authors:** Luciano Giardino, Eugenio Pedullà, Francesco Cavani, Francesca Bisciotti, Luca Giannetti, Vittorio Checchi, Daniele Angerame, Ugo Consolo, Luigi Generali

**Affiliations:** 1 Independent Researcher, 88900 Crotone, Italy; 2 Department of General Surgery and Surgical-Medical Specialties, University of Catania, 95131 Catania, Italy; eugeniopedulla@gmail.com; 3 Department of Biomedical Metabolic and Neural Sciences, Section of Human Morphology, University of Modena and Reggio Emilia, 41125 Modena, Italy; francesco.cavani@unimore.it; 4 Department of Surgery, Medicine, Dentistry and Morphological Sciences with Transplant Surgery, Oncology and Regenerative Medicine Relevance (CHIMOMO), University of Modena and Reggio Emilia, 41125 Modena, Italy; frabisciotti95@gmail.com (F.B.); luca.giannetti@unimore.it (L.G.); vittorio.checchi@unimore.it (V.C.); ugo.consolo@unimore.it (U.C.); 5 University Clinical Department of Medical, Surgical and Health Sciences, University of Trieste, 34149 Trieste, Italy; d.angerame@fmc.units.it

**Keywords:** chelators, confocal laser scanning microscope, dentinal tubules, surface tension, viscosity

## Abstract

This study aimed to examine the penetration depth into dentinal tubules of some chelating agents. The 17% EDTA and two preparations containing surfactants (Smear Clear, Bioakt Endo) were tested. Surface tension and liquid viscosity were measured using a Dynamic Contact Angle Analyzer and a Haake rotational rheometer. To measure the penetration depth inside dentinal tubules, thirty maxillary central incisors were selected from a pool of extracted human permanent teeth and allocated to three experimental groups (10 samples each), as well as were mechanically shaped and cleansed with 5.25% NaOCl, followed by each of the chelators being labeled with 0.1 wt % Rhodamine B according to final irrigation protocol established. The samples were embedded in an epoxy resin, after which 200 μm thick transverse sections were obtained at 2, 5, and 8 mm from the apex with a saw microtome. The specimens were then observed using a confocal laser microscope (CLSM) and the penetration of the labeled solution was measured in every third of each sample. Statistical analysis was performed using ANOVA or Kruskal–Wallis tests according to the distribution of data, evaluated with the Shapiro–Wilk normality test. Viscosity and surface tension tests have shown that BioAKT Endo has the lowest values compared to EDTA and Smear Clear. The medium penetration depth did not significantly differ among the three irrigants, while it increased considerably from the apical to the coronal level in all groups. Additionally, the maximum penetration depth increased significantly from the apical to coronal level, while among groups, BioAKT Endo showed the highest values at the apical and middle level compared to the other irrigants. No significant differences were observed among the three groups in medium and maximum penetration depths when the entire root was considered. New irrigants containing surfactants show reduced surface tension and, in one case (BioAKT Endo), viscosity. The lowering of the surface tension allows for better penetration of liquids into dentinal tubules than EDTA alone, thus improving the cleaning of the root canal system.

## 1. Introduction

The primary goal of endodontic therapy is to eradicate bacteria from the infected root canals and prevent their reinfection [1]. Matsuo et al. showed that bacteria are present in the dentinal tubules in 70% of endodontic infections [2]. Even with root canal instrumentation and disinfection, 65% of tubules remain infected [2], acting as an important reservoir of microorganisms for root canal reinfection [3]. In past years, Ando and Hoshino [4] have observed varying bacterial invasion depths within dentinal tubules, ranging from 500 to 2000 µm. Siqueira and Rôças, while studying debridement and bacteria remaining in the root canal, regardless of the instrumentation technique used, reported low debridement and inadequate disinfection of the root canal system [5]. The antibacterial effect, achieved by endodontic treatment, is more likely affected by the degree of the penetration of irrigants to scavenge bacteria residing deeply inside infected dentinal tubules than by the instrumentation of the root canal system because the shaping protocol revealed deficient debridement and areas untouched by both the manual K-files and rotary or reciprocating instruments [6]. Due to these limitations, in recent years, research on root canal irrigation quality and efficiency has focused on irrigating solutions with better cleaning and antibacterial activity as a necessary complement to mechanical preparation. Baker et al. [7] emphasized that the use of sodium hypochlorite (NaOCl) and ethylenediaminetetraacetic acid (EDTA) is the gold standard to dissolve necrotic tissue, remove the smear layer, and kill microorganisms in the root canal space. Nonetheless, due to their high surface tension, both irrigants do not deeply penetrate into dentinal tubules or other irregularities as isthmuses [8].

Although EDTA is the most commonly used chelator in contemporary endodontics, it has some drawbacks, such as a high surface tension [9], limiting its penetration into dentinal tubules; irregularities of the root canal system and isthmuses; and little or no antibacterial activity [10]. Conversely, a previous study reported that solutions with surface-active agents added are characterized by low surface tension [11], enhancing their antibacterial effect in line with other investigations [12,13]. One possible explanation for the increased bactericidal properties of irrigants is that incorporating different cleaning agents such as surfactants can enhance their wetting properties [14], hence their ability to penetrate more deeply into the dentinal tubules [15].

To overcome these problems, new irrigating solutions with detergents added, such as Smear Clear and more recently BioAKT Endo, have been introduced on the market to improve the clinical success rate of endodontic treatment. Smear Clear is a new class of 17% EDTA solution including a cationic cetrimide (CTR) and anionic surfactant Triton X-100, capable of lowering its surface tension by 32.5% compared to the original formula [6]. Recently, a new endodontic irrigant containing silver ions (0.003%) in citric acid (4.846%), specifically BioAKT Endo, was developed for clinical use to introduce a new two-in-one irrigant as an alternative to EDTA. Previous studies have reported that acidic solutions showed more effective smear layer removal [16] and improved antimicrobial activity in the root canal system [17] than EDTA-based solutions. Perhaps these results were due to their lower pH, which increased the removal of inorganic elements, such as calcium. Therefore, this study aimed to evaluate the surface tension and penetration depth into human dentin, and this new irrigant compared to other established solutions using a confocal laser scanning microscope (CLSM). The null hypothesis tested was that there are no significant differences in surface tension and both medium and maximum penetration depth into dentinal tubules among the irrigants employed. Moreover, preliminary laboratory measurements were performed on the three irrigants mentioned above to verify if their viscosity values could affect medium and maximum penetration depths into dentinal tubules.

## 2. Materials and Methods

This laboratory-based study evaluated the following solutions: 17% EDTA, Smear Clear, and BioAKT Endo (Table 1).

### 2.1. Surface Tension and Viscosity Measurements

Surface tension measurements, based on the geometry of the entirely wetted glass slide in contact with the liquids under investigation, were assessed using the Wilhelmy plate technique by the Cahn DCA-322 Dynamic Contact Angle Analyzer (Gibertini Elettronica, Novate (MI), Italy) as previously reported [18]. Each sample was measured five times to calculate the mean and standard deviation (SD). In addition, a preliminary pilot study was carried out on the three chelants to determine their viscosity using a Thermo Scientific RS100 Haake rotational rheometer (Thermo Fisher Scientific, Rodano, Italy) regulated at speeds of 100–1000 s^−1^ (https://www.drugfuture.com/Pharmacopoeia/EP7/DATA/20210E.PDF, accessed on 7 July 2021) [19]. Measurements were achieved at room temperature (21 °C) for each sample.

### 2.2. Sample Selection and Experimental Procedures

Thirty maxillary central incisors with single round-shape root canals with fully formed apices and straight canals between 0° and 5° were selected from a pool of extracted teeth and stored in a 0.1% thymol solution at 5 °C for no longer than 15 days. According to the Italian legislation, written informed consent for the use of teeth for research purposes was collected from all patients. The crown of each tooth was removed using a 701 high-speed fissure bur (Komet Italia, Milano, Italy) under water spray to obtain a working length (WL) at 14 ± 1 mm. Radiographs were taken in a mesiodistal and buccolingual plane with a size 10 K-file (Dentsply Maillefer, Baillagues, Switzerland) inserted into the canal to verify the straightening and select only teeth with a round canal, with a long:short cross-sectional diameter ratio of ≤2.5, 5 mm from the apex [20]. The working length (WL) and apical foramen patency were verified by insertion of a size 10 K-File (Dentsply Maillefer, Baillagues, Switzerland) until its tip appeared at the apical foramen under microscopic vision at 10× (OPMI Pico, Carl Zeiss Meditec Inc., Jena, Germany) [21], which then subtracting 0.5 mm. The specimens were divided into three experimental groups (n = 10 per group). The groups were homogeneous for canal width (Anova, P > 0.05) [22]. A mechanical glide path was performed with WaveOne Gold Glider reciprocating single files (size 0.15 and taper 0.017 to 0.085 at D16) (Dentsply Maillefer, Baillagues, Switzerland). Each root canal was instrumented with Wave One Gold Medium (size 0.35, taper 0.06) and then with WaveOne Gold Large (size 0.45, taper 0.07) (Dentsply Maillefer, Baillagues, Switzerland). The instruments were used in a slow in-and-out pecking motion mounted on an X-Smart plus endodontic engine (Dentsply Maillefer, Baillagues, Switzerland) set in the ”WAVE ONE ALL” mode up to 1 mm from the WL. Working length was re-checked with a size 10 K-File and eventually the instruments were used at full WL. At each instrument changeover, each canal was rinsed with 1 mL of 5.25% NaOCl (Niclor, Ogna, Muggiò, Italy) using a syringe with a 30-gauge side-vented needle (Max-i-Probe; Dentsply Rinn, Elgin, IL, USA) placed before the binding point but no closer than 2 mm to the WL. For each sample, a total of 10 mL of 5.25% NaOCl was used. The outer surface of the teeth was dried with paper towels and at the apical third, it was covered with a thin layer of dual composite (Opticore classic, IDS Spa, Savona, Italy).

In one group, the canals were finally rinsed with 5 mL of Smear Clear and in the others, with 5 mL of BioAKT Endo and with 5 mL of 17% EDTA: these irrigant solutions were labeled with 0.1% wt Rhodamine B dye (CARLO ERBA Reagenti S.r.l, Arese, Italy) and delivered using a syringe with a 25-gauge side-vented needle (Hawe Irrigation Probe, Kerr Corporation, Orange, CA, USA) placed at 2 mm from the WL. All chelating solutions were left in place for 3 min [23] and then the canals were dried with WaveOne Gold Large paper point (Dentsply, Maillefer, Baillagues, Switzerland).

Teeth were embedded in a room temperature-setting epoxy resin (Hard Rock 554, Remet, Bologna, Italy) and 200 μm thick transverse sections were obtained at 2, 5, and 8 mm from the apex with a saw microtome (Leica SP 1600, Nussloch, Germany). All sections were observed with a Leica TCS SP8 AOBS confocal microscope equipped with a White Light Laser (Mannheim, Germany) using the specific wavelengths for Rhodamine B and images were acquired using a 10× objective. Image mosaics were acquired and merged using the Navigator tool. Each image was obtained performing 20 μm z-stacks with one μm step size. The medium penetration depth of the irrigant solution into dentinal tubules was calculated as the average penetration measured at eight standardized points with the straight-line tool of the Fiji software (National Institutes of Health, Bethesda, MD, USA), starting from the inner side of the canal wall at 2, 5, and 8 mm level from the apex for each section [22]. The maximum penetration depth was measured as the deepest point of penetration from the canal wall at 2, 5, and 8 mm levels from the apex for each section, according to the observer [22].

### 2.3. Statistical Analysis

The normal distribution of all data obtained was evaluated with the Shapiro–Wilk normality test. When data were normally distributed, the comparison among groups and root levels within the same group were performed with the ANOVA test followed by Bonferroni’s post-hoc test. If not normally distributed, the non-parametric Kruskal–Wallis test followed by the Dunn test was used; p values < 0.05 were considered significant. Stata 11 software (StataCorp LLC, College Station, TX, USA) was used for the analysis.

## 3. Results

Our preliminary viscosity tests showed that BioAKT Endo had a viscosity value of 1.2 mPa s while Smear Clear and 17% EDTA had similar viscosities (2 mPa s) at 20 °C.

### 3.1. Surface Tension

Table 2 summarizes the surface tension evaluation results with the Wilhelmy plate technique. The 17% EDTA solution had the highest surface tension value compared to all other solutions (*p* < 0.001), while Bioakt Endo had the lowest one. In addition, a significant difference was observed between BioAKT Endo and Smear Clear (*p* < 0.05).

### 3.2. Penetration Depth into Dentinal Tubules

Means and standard deviations relative to the irrigant medium penetration are reported in Table 3. No significant differences could be found among the three irrigants employed when the same root levels were compared or even when considering the whole root altogether; nonetheless, BioAKT Endo showed the highest values among the three groups. Within each group, a significant increase in the medium penetration depth was observed from apical to coronal sections.

**Figure 1:**
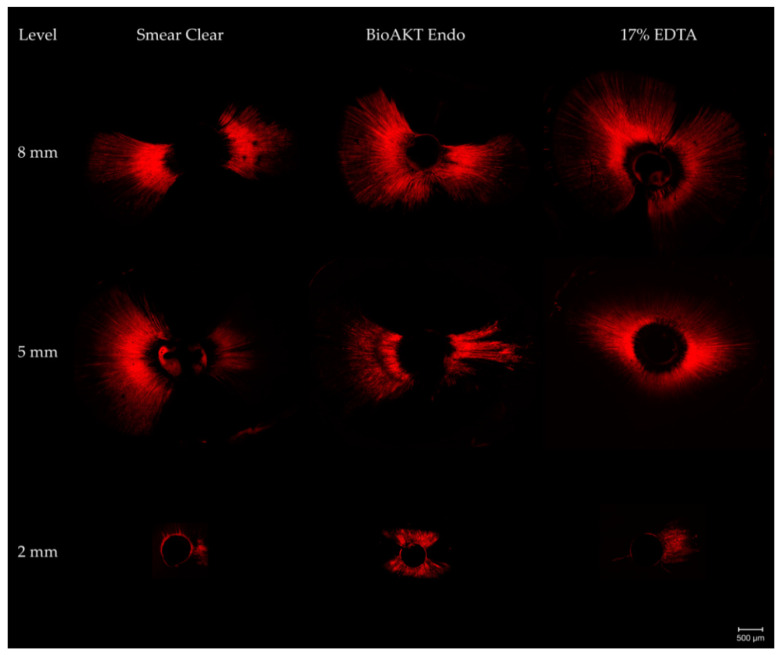
Representative images of the evaluated groups.

Figure 1 shows an overview of representative CLSM images from three experimental groups at 2, 5, and 8 mm levels.

As for the maximum penetration depth, data are reported in Table 4. At the apical level, the BioAKT Endo group had the highest values compared to the other groups. In particular, it was significantly higher than the EDTA values.

At the middle level, both the BioAKT Endo and Smear Clear groups showed significantly higher maximum penetration depths with respect to that of the EDTA. No significant differences were recorded at the coronal level among groups. When the entire root was considered, no significant differences were observed among the three groups regarding the maximum penetration depth. The maximum penetration depth increased significantly from the apical to the coronal level in each group.

## 4. Discussion

In the present study, the surface tension and penetration depth of some irrigating solutions containing EDTA or citric acid were evaluated inside dentine tubules in human teeth.

Smear Clear is a 17% EDTA irrigant containing the cationic detergent cetrimide (CTR) and one additional non-ionic surfactant Triton X-100. BioAKT Endo, instead, is a silver citrate solution wherein silver ions are weakly bonded to citric acid. Besides a good ability to remove the smear layer, both solutions demonstrated significant antibacterial activity [12,24,25]. Previous reports highlighted that incorporating detergents into irrigants could reduce their surface tension, enhance the solution’s wettability, and improve the root canal system disinfection [26]. Wettability has been defined as the tendency of a fluid to spread over or adhere to a solid surface as dentin. This feature is necessary for the irrigant to penetrate the root canal system cavities, depending on the surface tension [27]. In the present study, we found that the surface tension values of both 17% EDTA and citric acid-based chemical agents with detergents added were lower than the 17% EDTA alone (Table 1), confirming previous investigations [8,25]. The null hypothesis concerning the surface tension of the irrigants tested must be rejected as BioAKT and Smear Clear showed a lower surface tension than EDTA. Conversely, the penetration depth of the chelators mentioned above have not yet been evaluated. For this reason, this investigation was mainly designed to assess the penetration depth into dentinal tubules of the chemical agents herein used as final irrigating solutions. To date, there is only one investigation concerning the sealer penetration of silver citrate solutions [24], in which the labeled sealer with a fluorescent dye was used as an indicator of sealer-filled root canal dentin treated with the silver citrate solution compared to other irrigants. Instead, no studies are available in the literature concerning the penetration depth of these new chelating agents into dentinal tubules. Our results indicate higher penetration depths in the coronal and middle thirds of specimens from all the groups; additionally, a lower penetration was seen in the apical third of all the root canals analyzed (Table 2). As for medium penetration, since no significant differences emerged among the irrigants, the null hypothesis must be accepted, although it must also be rejected for the maximum penetration depth as BioAKT Endo and Smear Clear had significantly better performances. Current results could be explained by the fact that the anatomical structure of the root canal system can influence irrigants’ penetration depth into dentinal tubules. Indeed, dentinal tubules in the apical third of the root canals are less permeable than those in the coronal and middle thirds due to tubular sclerosis, the smaller diameter, and the reduced number [28]. After the age of 30, dentinal sclerosis develops, starting from the apical third of teeth leading to the “butterfly effect” [22,29]. This optical effect leads to the obliteration (sclerosis) of most dentinal tubules in the mesial and distal directions. The great variability observed probably depends on the age of patients that were not selected according to a specific age range; on the contrary, age was unknown. This fact, strictly linked to the butterfly effect, could explain the great variability and standard deviation observed in all the groups and regions analyzed. Furthermore, it was highlighted that the constriction of the root canal apical third hinders the flow and backflow of irrigating solutions, compromising its cleaning [30], probably because the narrowing would inhibit the needle placement inside the apical third and the resultant fluid dynamics [31]. BioAKT Endo showed greater medium penetration depth, although not significantly, than 17% EDTA and Smear Clear in all the thirds of the root canals (Table 3). These results could be explained by the chelators’ different surface tensions, viscosities, molecular weights, and pH values. EDTA showed a significantly higher surface tension value (Table 2) and a lower maximum penetration depth (Table 4) than other irrigant solutions. As for the similar penetration recorded at the apical level in both the EDTA and Smear Clear groups, it must be underlined that their similar viscosity could have greatly affected the penetration in this area of the root where dentinal sclerosis greatly reduces the diameter of the tubules. In fact, besides surface tension, an essential aspect of irrigant flow is its viscosity, affecting the irrigant penetration and its flow within the root canal [32]. Studies have shown increased tubular penetration with decreased viscosity and surface tension [32]. According to the present study results, BioAKT Endo (citric acid-based chelator) viscosity was lower than both EDTA solutions, regardless of the presence or absence of detergents. The viscosity of a solution is related to its molecular weight, affecting the liquid flow and its penetration in dentinal tubules [33,34]. With its lower molecular weight (https://pubchem.ncbi.nlm.nih.gov/compound/Citric-acid, accessed on 7 July 2021), citric acid can easily penetrate deeper into dentinal tubules than EDTA, the highest molecular weight chelator (https://pubchem.ncbi.nlm.nih.gov/compound/EDTA-disodium-salt, accessed on 7 July 2021). Furthermore, larger molecules as EDTA bind to fewer calcium ions in dentin [35]. Concerning the action of each solution related to its composition and pH, a report highlighted that using a low concentration (5%) of citric acid at acidic pH, similar to the BioAKT Endo composition, is adequate to remove the smear layer as efficiently as done for higher concentrations [36]. Moreover, Poggio et al. [37] compared the decalcifying capacity of different irrigating solutions at different contact times, observing a significantly higher release of calcium (Ca^++^) in samples exposed to citric acid-based agents than that of EDTA solutions. The higher release reported in that study may be related to the lower pH of the citric acid solutions (pH < 2), thus increasing the removal of inorganic elements such as Ca^++^ from the hydroxyapatite crystals. The addition of detergents did not affect the extraction properties of EDTA and citric acid solutions because the values of the concentration of Ca^++^ released in the two solutions did not significantly differ. Therefore, the addition of surfactants to EDTA (i.e., Smear Clear) does not seem to alter its viscosity, although it has a profound effect on surface tension, which is extremely reduced, making it similar to BioAKT Endo. Sousa and Silva [38] have shown that 1% citric acid solution at pH 1.0 removed more calcium than pH 7.4. Conversely, there were no differences between 1% citric acid at pH 7.4 and saline solution, which had the least efficacy for Ca^++^ extraction. Citric acid monopolizes such a large portion of the solvent at high concentrations that the amount of solvent available for Ca^++^ diffusion is dramatically reduced [38]. Then, the pH of the citric acid solution becomes a more important factor in demineralization than concentration [39]. The lower ability of EDTA compared to citric acid in removing the smear layer can be explained by the fact that EDTA properties are self-limiting due to its neutral pH [40]. Since 99% of EDTA exists as EDTAHNa_3_ (trisodium EDTA), the exchange of Ca^++^ from dentin will occur by H^+^ ions and subsequently decrease in pH, hence increasing in acidity; therefore, the effect of EDTA decreases with the increase in acidity. Unlike EDTA, acidic chelators instead depend on the hydrogen ion concentration for their demineralization effects as organic acids [35]. Pending further clinical and safety studies, other studies on these new chelators combined with detergents should be undertaken to confirm the efficacy and safety of these agents for root canal therapy in daily clinical practice.

## 5. Conclusions

Under these experimental conditions and within the limits of the present study, BioAKT and Smear Clear showed a lower surface tension than EDTA. As for the medium penetration, no significant differences emerged among the irrigants, while BioAKT Endo and Smear Clear had significantly better performances than EDTA at the apical and middle levels for maximum penetration depth. The presence of surfactants, lowering the surface tension of irrigants, can improve their maximum penetration, irrespective of their viscosity value. These characteristics should be considered to enhance the cleaning of root canal walls and dentinal tubules.

## Figures and Tables

**Table 1:** Chelating agents’ compositions and manufacturers.

Chelating Agents	Compositions	Manufacturer	
EDTA	17% ethylenediaminetetraacetic acid water	Ogna Laboratori Farmaceutici, Muggio, Italy	
Smear Clear	17% EDTA Cetrimide (% unknown) Triton X-100 (% unknown) water	KerrHawe SA, Bioggio, Switzerland	
BioAKT Endo	4.846% citric acid 0.003% silver ions Detergents unknown water	New Tech Solutions s.r.l., Brescia, Italy	

**Table 2:** Surface tension values (mJ/m^2^) of the irrigating solutions.

Solutions	Number of Samples	Surface Tension Mean Value ± SD
17% EDTA	5	* 48.9 ± 0.65
Smear Clear	5	33.00 ± 0.62
BioAKT Endo	5	** 32.00 ± 0.59

* *p* < 0.001 vs. BioAKT Endo and Smear Clear; ** *p* < 0.05 vs. Smear Clear (Kruskal–Wallis).

**Table 3:** Medium penetration of irrigants (µm, mean ± s.d.).

Sample Level	Smear Clear	BioAKT Endo	EDTA
Apical	82 ± 89	172 ± 229	63 ± 75
Middle	361 ± 183 *	606 ± 527 *	516 ± 404 *
Coronal	911 ± 420 *^,^**	1067 ± 485 *^,^**	952 ± 313 *^,^**
Total	451 ± 436	615 ± 560	511 ± 468

* *p* < 0.05 vs. apical of same group; ** *p* < 0.05 vs. middle of same group (Kruskal–Wallis).

**Table 4:** Maximum penetration of irrigants (µm, mean ± s.d.).

Sample Level	Smear Clear	BioAKT Endo	EDTA
Apical	329 ± 240	508 ± 459 ^§^	129 ± 147
Middle	1339 ± 517 ^§§,^*	1351 ± 822 ^§§,^*	874 ± 458 *
Coronal	2022 ± 439 *^,^**	1942 ± 617 *	1543 ± 300 *^,^**
Total	1230 ± 813	1267 ± 867	849 ± 666

^§^ *p* < 0.05 vs. EDTA apical; ^§§^ *p* < 0.05 vs. EDTA middle (ANOVA). * *p* < 0.05 vs. Apical of the same group; ** *p* < 0.05 vs. middle of same group (Kruskal–Wallis).

## Data Availability

The data presented in this study are available on request from the corresponding author.
